# IGF2 prevents dopaminergic neuronal loss and decreases intracellular alpha-synuclein accumulation in Parkinson’s disease models

**DOI:** 10.1038/s41420-023-01734-1

**Published:** 2023-12-02

**Authors:** Javiera Arcos, Felipe Grunenwald, Denisse Sepulveda, Carolina Jerez, Valentina Urbina, Tomas Huerta, Paulina Troncoso-Escudero, Daniel Tirado, Angela Perez, Rodrigo Diaz-Espinoza, Esteban Nova, Ulrich Kubitscheck, Juan Eduardo Rodriguez-Gatica, Claudio Hetz, Jorge Toledo, Pablo Ahumada, Diego Rojas-Rivera, Elisa Martín-Montañez, María Garcia-Fernandez, René L. Vidal

**Affiliations:** 1 Center for Integrative Biology, Universidad Mayor, Santiago, Chile; 2 Biomedical Neuroscience Institute, University of Chile, Santiago, Chile; 3 Center for Geroscience, Brain Health and Metabolism, Santiago, Chile; 4 Molecular Diagnostic and Biomarkers Laboratory, Department of Pathology, Faculty of Medicine Clínica Alemana, Universidad del Desarrollo, Santiago, Chile; 5 Escuela de Tecnología Médica, Universidad Mayor, Santiago, Chile; 6 Departamento de Biología, Facultad de Química y Biología, Universidad de Santiago de Chile, Santiago, Chile; 7 Departamento de Química, Facultad de Ciencias Naturales, Matemáticas y Medio Ambiente, Universidad Tecnológica Metropolitana, Santiago, Chile; 8 Clausius Institute of Physical and Theoretical Chemistry, University of Bonn, Bonn, Germany; 9 Escuela de Biotecnología, Universidad Mayor, Santiago, Chile; 10 Center for Biomedicine, Universidad Mayor, Santiago, Chile; 11 Department of Pharmacology, Faculty of Medicine, Biomedical Research Institute of Malaga, University of Malaga, Malaga, Spain; 12 Department of Human Physiology, Faculty of Medicine, Biomedical Research Institute of Malaga, University of Malaga, Malaga, Spain

**Keywords:** Parkinson's disease, Parkinson's disease

## Abstract

Parkinson’s disease (PD) is the second most common late-onset neurodegenerative disease and the predominant cause of movement problems. PD is characterized by motor control impairment by extensive loss of dopaminergic neurons in the substantia nigra pars compacta (SNpc). This selective dopaminergic neuronal loss is in part triggered by intracellular protein inclusions called Lewy bodies, which are composed mainly of misfolded alpha-synuclein (α-syn) protein. We previously reported insulin-like growth factor 2 (IGF2) as a key protein downregulated in PD patients. Here we demonstrated that IGF2 treatment or IGF2 overexpression reduced the α-syn aggregates and their toxicity by IGF2 receptor (IGF2R) activation in cellular PD models. Also, we observed IGF2 and its interaction with IGF2R enhance the α-syn secretion. To determine the possible IGF2 neuroprotective effect in vivo we used a gene therapy approach in an idiopathic PD model based on α-syn preformed fibrils intracerebral injection. IGF2 gene therapy revealed a significantly preventing of motor impairment in idiopathic PD model. Moreover, IGF2 expression prevents dopaminergic neuronal loss in the SN together with a decrease in α-syn accumulation (phospho-α-syn levels) in the striatum and SN brain region. Furthermore, the IGF2 neuroprotective effect was associated with the prevention of synaptic spines loss in dopaminergic neurons in vivo. The possible mechanism of IGF2 in cell survival effect could be associated with the decrease of the intracellular accumulation of α-syn and the improvement of dopaminergic synaptic function. Our results identify to IGF2 as a relevant factor for the prevention of α-syn toxicity in both in vitro and preclinical PD models.

## Introduction

Parkinson’s disease (PD) is the second most common late-onset neurodegenerative disease and the predominant cause of movement disabilities [1, 2]. Its incidence increases with age, affecting 0.6% of the population between 65 and 69 years old and 2.6% of people from 85–89 years of age [3]. PD is characterized by motor control impairment that results from the extensive loss of dopaminergic neurons in the substantia nigra pars compacta (SNpc) and formation of protein inclusions called Lewy bodies, which are composed mainly of α-synuclein (α-syn) protein aggregates [4, 5]. Despite the numerous studies available in both cellular and animal models of PD, as well as in human PD-derived tissue, the molecular mechanisms involved in the selective neurodegeneration observed in PD are just starting to be elucidated. To date, many different stress pathways have been implicated in the disease process, importantly including the impairment of the proteostasis network [6, 7]. We have reported that protein folding stress at the level of the endoplasmic reticulum (ER) contributes to dopaminergic neuron loss in pharmacological models of PD in vivo [8]. A dynamic signaling network known as the unfolded protein response (UPR) is involved in the restoration of the protein homeostasis imbalance. Previously, we have uncovered a relevant role for XBP1 a conserved branch of the UPR in the progression of PD [8], and also for Huntington’s disease (HD) [9], using a conditional knockout mouse model for XBP1 in the central nervous system. In these studies, XBP1 deficiency triggered significant neuroprotection by a mechanism involving upregulation of autophagy, reducing abnormal protein aggregation which led to a delay in disease progression at the motor and histological level [9].

To define the possible mechanism explaining the neuroprotective effects detected in XBP1 deficient animals, we performed an unbiased screening of the gene expression profile of SN brain tissue using Micro Arrays followed by confirmation using real time PCR. From the global analysis of this data, we observed to insulin-like growth factor 2 (IGF2) as a major hit upregulated in XBP1 knockout brains [10]. A network of trophic relationships maintains neuronal viability, not only during development but also in adult life. An ever-increasing number of humoral growth factors known to be active in different tissues are also implicated in brain physiology. While recognized for decades as neuroactive, insulin and its relatives, IGF1 and IGF2, have not been categorized as neurotrophic peptides [11]; however, they can affect neuronal homeostasis through different mechanisms of action [12].

IGF2, the less studied insulin-related peptide, is a single-chain polypeptide that belongs to a family of insulin-related proteins, is expressed in most tissues during fetal life, and it is found predominantly within the brain and spinal cord during adulthood [13, 14]. In vitro studies have described IGF2 as a survival factor in neurons [15, 16]. IGF2 exhibits a higher affinity for the IGF2 receptor (IGF2R), although it can also bind with lower affinity to IGF1R and cause the downstream activation of typical tyrosine kinase-mediated signaling pathways [17]. Several reports have shown that IGF2 is involved in neurophysiological processes. For example, *Igf2* is a target of the transcription factor C/EBPβ that is engaged during memory consolidation in rats [18], and IGF2 overexpression was shown to rescue working memory deficits in a schizophrenia mouse model [19]. In addition, IGF2 has been described as a regulator of adult hippocampal neurogenesis [20].

The expression of insulin, IGF1, IGF2 and its receptors and downstream substrates are reduced in the brain of Alzheimer’s disease (AD) patients [21–23]. These alterations have been associated with a resistance to IGF1 stimulation, reflected in an attenuation of downstream AKT and ERK signaling as demonstrated in hippocampal brain slices from AD patients [24, 25]. Treatment with insulin and/or IGF1 have been shown to protect neurons against amyloid β-induced neurotoxicity, enhancing memory performance of AD patients [26] and rodents in preclinical models of AD [27–30]. Moreover, the intracerebroventricular injection of IGF2 reduced the number of amyloid β plaques in the hippocampus of an AD mouse model [31]. IGF2 treatment also reduced the occurrence of several pathological processes associated with AD as amyloidosis, and increase of cholinergic marker [31].

We recently describe the neuroprotective effect of IGF2 in Huntington’s disease (HD) [10]. Cell culture studies demonstrate that IGF2 expression reduces the load of intracellular aggregates of mutant huntingtin (mHtt) and a polyglutamine peptide (polyQ), which was associated with a decrease in the half-life of the proteins. Importantly, IGF2 treatment protected medium spiny neurons (MSNs) derived from HD patients, in addition to spinocerebellar ataxia 3 (SCA3) patients, the second most common polyQ disease [10]. Administration of an IGF2 gene therapy into the striatum resulted on a marked decrease in the levels of mHtt aggregation in three different animal models of HD. Moreover, a marked reduction of IGF2 protein levels caudate-putamen samples from HD patients when compared with healthy donors. Also, IGF2 protein levels diminished in peripheral blood mononuclear cells from HD patients [10].

A few studies have associated the impairment of insulin/IGF1 signaling to the physiopathology of PD. In vitro studies have shown that treatment of neurons with IGF1 reduces the toxicity associated with altered dopamine secretion in primary cultures [32]. Moreover, IGF1 protects cells against the PD-inducing neurotoxin, associated with the activation of glycogen synthase 3 kinase (GSK3) [33], and the reduction in the levels of α-syn aggregation through activation of the phosphoinositide 3 kinase (PI3K) and AKT pathway [34]. A protective effect of IGF1 was also reported in PD models in vivo. Treatment with recombinant IGF1 by intraperitoneal injection significantly reduced the loss of dopaminergic neurons in the SNpc and improved functional deficits in animals injected with a PD-inducing neurotoxin in the striatum [35, 36]. These effects of IGF1 into PD model were blocked by inhibition of PI3K/AKT signaling [37]. Other reports have also validated the prosurvival activity of IGFs, where it is signaling blocks the proapoptotic function of GSK3 and the transcription factors of the Forkhead box O (FOXO) family [38, 39]. The activation of the IGF1/AKT pathway also protects neurons against experimental HD possibly mediated by an increased clearance of mHtt by autophagy [40, 41]. Remarkably, recent genetic linkage studies have associated IGF2 with PD. A polymorphism in the *IGF2* gene was identified as a possible modifier of the susceptibility to develop idiopathic PD in a Caucasian group from Brisbane, Australia [42]. This polymorphism was also associated with lower body weight and alteration of the tyrosine hydroxylase (TH) enzyme expression that is engage in the dopamine production [43]. Interestingly, low body mass index, potentially reflecting a broader metabolic disorder, is observed in PD patients [44, 45]. Moreover, our laboratory has recently reported a significant decrease of IGF2 levels in both plasma and peripheral blood mononuclear cells (PBMCs) from Chilean PD patients [46].

Taken together, accumulating evidence suggests a relevant role of IGF2 in the global control of homeostasis in the brain, which may impact neurodegenerative events in PD. We postulate that changes in IGF2 levels and/or IGF2 signaling could prevent to the differential neuronal vulnerability of dopaminergic neurons, motor impairment and α-syn accumulation in PD and arise as a potential neuroprotective factor to prevent the dopaminergic neuronal loss on PD.

## Results

### IGF2 treatment prevent the cytotoxicity trigger by α-syn PFF in PD cellular models

IGF2, is a secreted factor which has been recently recognized with interesting neuroprotective activities in several models of neurodegenerative diseases, including AD, Amyotrophic Lateral Sclerosis and HD. Nevertheless, less evidence is available about the possible contribution of IGF2 in the survival of dopaminergic neurons and its role in the PD progression.

**Fig. 1:**
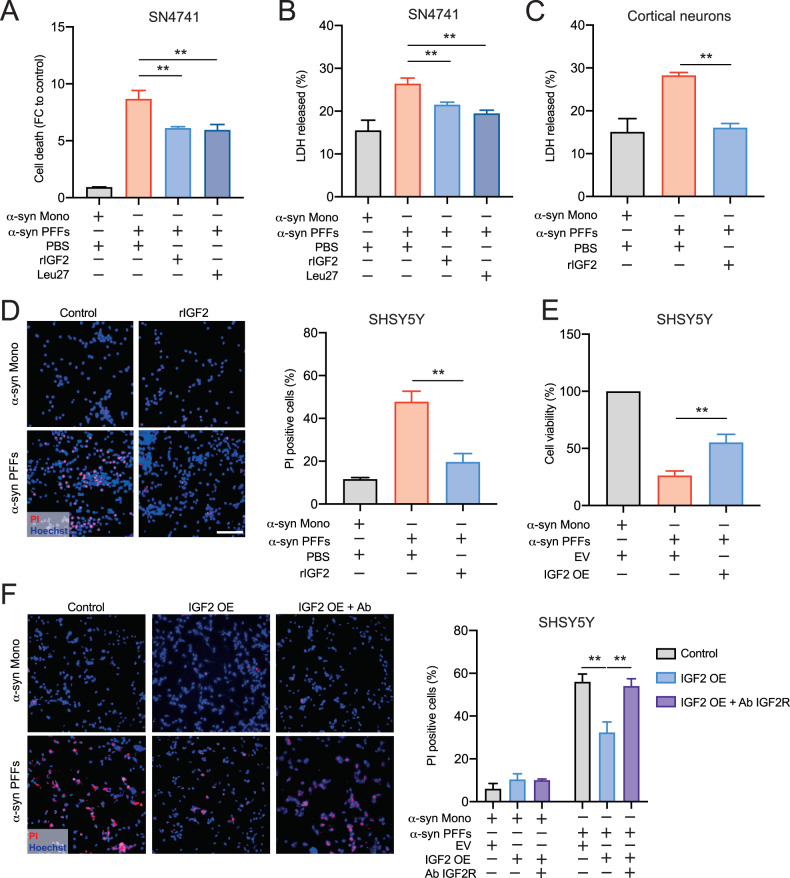
IGF2 expression prevents cytotoxicity effect of α-syn PFF in PD cellular models. **A**, **B** SN4741 cells were treated with human recombinant IGF2 (rIGF2) 5 ng/ml or Leu27 5 ng/ml for 1 h. and exposed to human α-syn monomers (Mono) or α-syn PFF. PBS 1× was used as a control. After 24 h, cell death was measured by Sytogreen (**A**) and LDH assay (**B**). FC correspond to fold change. **C** Primary culture of cortical neurons was treated at 6 DIV with rmIGF2 100 ng/mL 1 h previously to incubation with mouse α-syn monomers and α-syn PFF. PBS 1× was used as a treatment control. After 72 h of incubation, cellular damage was evaluated by LDH released assay. **D** SHSY5Y cells were treated with human recombinant IGF2 (rIGF2) 5 ng/ml for 1 h and exposed to human α-syn monomers and α-syn PFF. After 24 h, cell death was measured by quantification of PI-positive cells. **E**, **F** SHSY5Y cells were transfected with IGF2-containing plasmid to overexpress IGF2 (IGF2 OE) or empty vector (EV) as control. After 24 h cells were incubated with human α-syn monomers or α-syn PFF and IGF2 receptor antibody (IGF2 OE + Ab IGF2R). Followed 24 h, cell viability was measured by cresyl violet staining (**E**) and by quantification of PI-positive cells (**F**). Scale bar 100 um. Data are presented as mean and SEM of at least three independent experiments. Statistically significant differences detected by two-tailed unpaired *t* test (**p < 0.01).

PD is characterized by the formation of protein inclusions called Lewy bodies, which are composed mainly of α-syn protein aggregates and ubiquitinated proteins [4, 5]. The abnormal α-syn accumulation has been associated with protein imbalance and consequently, it triggers the selective neuronal loss. Several studies demonstrate that incubation of α-syn amyloid form in neuronal cell trigger cellular toxicity leading to neurodegeneration like PD. Therefore, to evaluate the IGF2 effect on α-syn-induced cytotoxicity, we performed IGF2 gain-of-function in cellular PD model based on the α-syn preformed fibrils (α-syn PFF) toxicity. First, we analyzed the effect of IGF2 signaling using human recombinant IGF2 (rIGF2) in mouse embryonic substantia nigra-derived neural cell line (SN4741 cells) and it was observed a significant reduction of cellular toxicity trigger by α-syn PFF under rIGF2 treatment (Fig. 1A, B). Considering a possible effect of rIGF2 on IGF1R and insulin receptor, we used a synthetic peptide called Leu-27 which stimulate specially IGF2 receptor (IGF2R) [47]. We observed a neuroprotective effect of Leu-27 peptide against α-syn PFF toxicity in SN4741 cells (Fig. 1A, B). Then, we evaluate the IGF2 effect in cortical primary neurons treated with α-syn PFFs which produce α-syn intracellular inclusions and toxicity [48, 49]. Cortical neurons cultured for 7 days were co-treated with rIGF2 and α-syn PFF for 7 days. We achieved an increase of LDH release trigger by α-syn PFF which was diminished by rIGF2 treatment (Fig. 1C). To complement these results, we used human neuroblastoma cell line (SHSY5Y cells) treated with α-syn PFF. rIGF2 treatment decreases the cytotoxicity trigger by α-syn PFF in SHSY5Y (Fig. 1D).

To determine the impact of IGF2 gain-of function, we evaluated the possible neuroprotective effect of IGF2 overexpression (IGF2 OE) in cellular PD model. IGF2 OE prevented cell death in SHSY5Y cells expose to α-syn PFF (Fig. 1E, F). Additionally, we determine the participation of the IGF2 receptor (IGF2R) in the neuroprotective effect of IGF2 OE. We blocked the IGF2 receptor using anti-IGF2R antibody, and we observed a significantly delaying of the IGF2 neuroprotective effect in SHSY5Y cells expose to α-syn PFF (Fig. 1F).

These results showed that incubation with α-syn PFFs decrease cell viability and rIGF2 or IGF2 OE treatment significantly prevents the α-syn PFF cytotoxicity in murine and human PD cellular models.

### IGF2 treatment prevents the α-syn intracellular accumulation and promotes the α-syn secretion in PD cellular models

**Fig. 2:**
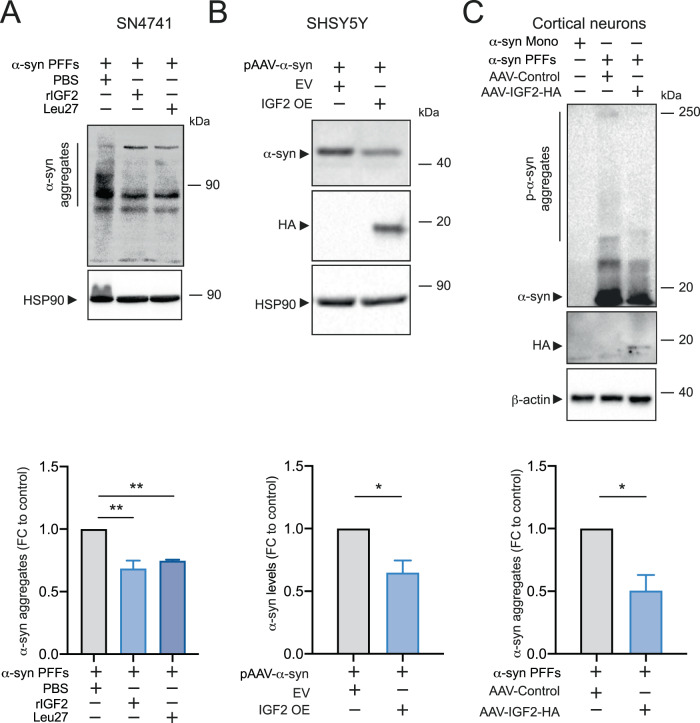
IGF2 expression decrease α-syn aggregates in PD cellular models. **A** SN4741 cells were treated with human recombinant IGF2 (rIGF2) 5 ng/ml or Leu27 5 ng/ml for 1 h and exposed to α-syn monomers (Mono) or α-syn PFF. PBS 1× was used as a control. After 24 h, α-syn levels were evaluated in whole cells extracts by western blot (left panel), using anti-α-syn antibody (upper panel). HSP90 levels were monitored as loading control (bottom panel). Bottom panel. α-syn levels were quantified and normalized to HSP90 levels. **B** SHSY5Y cells were transiently co-transfected with pAAV-α-syn-RFP expression vector and pAAV-IGF2-HA vector (IGF2 OE) or pAAV-empty vector (EV). 48 h later, α-syn levels were analyzed in whole cells extracts by western blot analysis using anti-α-syn antibody (upper panel). IGF2 levels were also determined (middle panel). Hsp90 levels were monitored as loading control (bottom panel). Bottom panel. α-syn levels were quantified and normalized to HSP90 levels. **C** Primary culture of cortical neurons 1 DIV were infected with AAV-IGF2 or AAV-control. 7 days later, neurons were treated with PFF or monomer (control) of α-syn. 7 days later, α-syn levels were evaluated in whole cells extracts by western blot (left panel) using anti-α-syn phosphorylated antibody (upper panel). IGF2 levels were determined (middle panel). b-actin levels were monitored as loading control (bottom panel). Bottom panel. α-syn levels were quantified and normalized to actin levels. Data are presented as mean and SEM of at least three independent experiments. Statistically significant differences detected by two-tailed unpaired *t* test (*p < 0.05, **p < 0.01).

To evaluate the possible mechanism involved in the IGF2 neuroprotective effect, we monitored α-syn intracellular accumulation levels. SN4741 cells were treated with α-syn PFF and rIGF2. rIGF2 treatment showed a significant reduction of α-syn aggregation levels in SN4741 cells. Moreover, the Leu-27 treatment trigger also a diminished of α-syn intracellular aggregation levels in SN4741 cells (Fig. 2A). Additionally, we observed a diminished of α-syn intracellular levels by IGF2 OE in SHSY5Y cells transiently transfected with α-syn and IGF2-HA vector (Fig. 2B). We also assessed the impact of IGF2 OE on α-syn intracellular accumulation in cortical primary neurons. We transduced cortical neurons with adeno-associated virus to overexpress IGF2-HA (AAV-IGF2-HA) at 1 day in vitro (1 DIV) and after 7 days these cells were treated with α-syn PFF by 7 days. An empty AAV was used as control (AAV-Control). We observed a significantly decrease of α-syn aggregation levels for IGF2 OE in cortical neurons treated with α-syn PFF (Fig. 2C).

**Fig. 3:**
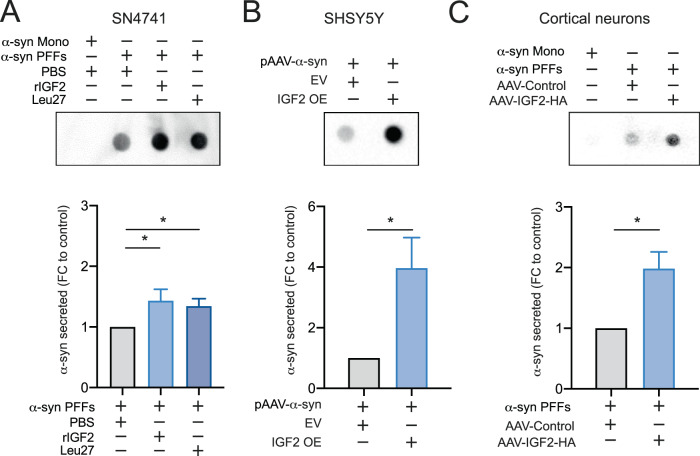
IGF2 expression increase α-syn secretion in PD cellular models. **A** SN4741 cells were treated with human recombinant IGF2 (rIGF2) 5 ng/ml or Leu27 5 ng/ml for 1 h and exposed to α-syn monomers (Mono) or α-syn PFF. PBS 1× was used as a control. After 24 h, culture media was analyzed by dot blot using an anti-α-syn antibody (upper panel). α-syn levels were quantified and plotted in each condition (bottom panel). **B** SHSY5Y cells were transiently co-transfected with pAAV-α-syn-RFP expression vector and pAAV-IGF2-HA vector (IGF2 OE) or pAAV-empty vector (EV). After 48 h culture media was analyzed by dot blot using an anti-α-syn antibody (upper panel). α-syn levels were quantified and plotted in each condition (bottom panel). **C** Primary culture of cortical neurons (1 DIV) were infected with AAV-IGF2 or AAV-control. 7 days later, neurons were treated with PFF or monomer (Mono) of α-syn. 7 days later, α-syn levels was analyzed by dot blot using an anti-α-syn antibody (upper panel). α-syn levels were quantified and plotted in each condition (bottom panel). Data are presented as mean and SEM of at least three independent experiments. Statistically significant differences detected by two-tailed unpaired *t* test (*p < 0.05).

We explored the possibility that the α-syn protein was redirected to another subcellular compartment or released outside of the cell. Although α-syn is a cytosolic protein with a predominant localization in presynaptic terminals [50], it is also found outside the cell [51]. In fact, extracellular fractions of soluble α-syn have been detected in the cerebrospinal fluid (CSF) and plasma samples from healthy individuals and PD patients [52, 53]. To determine the impact of IGF2 treatment on α-syn secretion, we monitored its presence in the cell culture media. Under resting conditions, we could detect α-syn outside the cell using dot blot assay. SN4741 cells were treated with α-syn PFF and rIGF2. rIGF2 treatment showed a significant increase of α-syn secretion levels in SN4741 cells (Fig. 3A). Moreover, the Leu-27 treatment trigger also a diminished of α-syn secretion levels in SN4741 cells (Fig. 3A). We also observed an increase of α-syn secreted in SHSY5Y cells transiently transfected with α-syn and IGF2-HA vector (Fig. 3B). To complement these results, we evaluated the α-syn secretion in cortical neurons treated with α-syn PFF. We observed an increase of α-syn secretion in cortical neurons trigger by IGF2 OE (Fig. 3C).

These results demonstrate the neuroprotective effect of IGF2 on cellular toxicity could be explained by the decrease of intracellular α-syn levels and increase of its secretion trigger by IGF2 treatment. Moreover, our results suggest a participation of IGF2R in this phenomenon.

Based on these observations, we explorer the IGF2 neuroprotective effect in a PD mouse model based on intracerebral injection of α-syn PFFs.

### IGF2 prevents dopaminergic neuron degeneration in PD idiopathic model

**Fig. 4:**
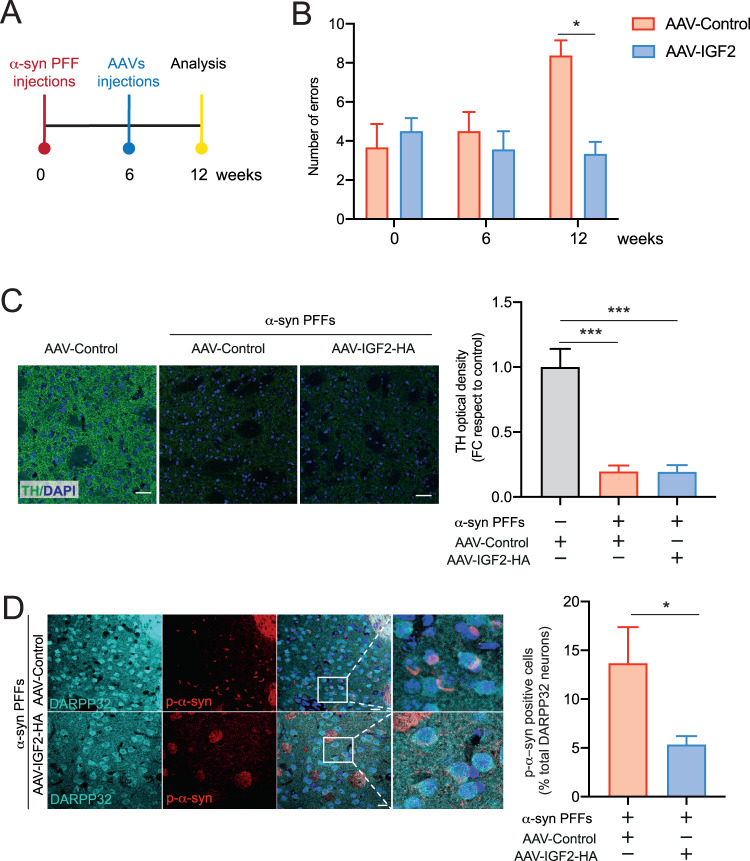
IGF2 treatment improved motor performance in an idiopathic PD mice model. **A** Experimental strategy to determine the effect of IGF2 gene therapy in idiopathic PD mice model. **B** Beam test was performed to evaluate spontaneous motor changes associated with dopamine depletion in the striatum of α-syn PFF injected mice. Number of errors for steps was measure and plotted for each condition at basal condition (0 weeks), 6, or 12 weeks. **C** Dopaminergic innervation was detected using anti-TH antibody (green), in striatum tissue. Total cells were visualized using DAPI staining (blue). FC correspond to fold change. Scale bar = 20 μm. **D** Medium spine neuron present in the striatum brain region was visualized using anti-DARPP32 antibody (capyso). Phospho -α-syn (p-α-syn) accumulation were visualized in striatum tissue sections by anti-p-α-syn immunostaining (red). Total cells were visualized using DAPI staining (blue). Scale bar = 20 μm. For all experiment mean and SEM were represented for seven independent experiments. Statistically significant differences were detected by two-tailed unpaired *t* test (*p < 0.05).

We generated an idiopathic model of PD based on the α-syn PFFs unilateral intracerebral injection in the striatum brain region. After 6 weeks of α-syn PFFs injection, we performed injections of AAVs to induce the expression of IGF2 in dopaminergic neurons of the SNpc region (Fig. 4A) and later 6 weeks we evaluated it possible neuroprotective effect in PD in vivo model. First, we evaluate the possible IGF2 therapeutic effect on motor impairment observed in this PD model. We determine the motor coordination activity using beam test [54] and we observed that AAV-IGF2 gene therapy prevents motor dysfunction in the idiopathic PD model (Fig. 4B). An early event observed in PD is axonal degeneration, leading to the loss of innervation of striatal neurons before dopaminergic neuron death. For this reason, we investigated the possible alteration in dopaminergic innervation on striatum region. We evaluated the TH, as a marker for dopaminergic innervation in the striatum. We observed similar dopaminergic innervation levels in idiopathic PD model treated with IGF2 therapy compared to the control group (Fig. 4C). However, AAV-IGF2 gene therapy treatment significantly prevented the accumulation of phosphorylated α-syn (p-α-syn) on DARPP32 positive neurons in the striatum region (Fig. 4D).

**Fig. 5:**
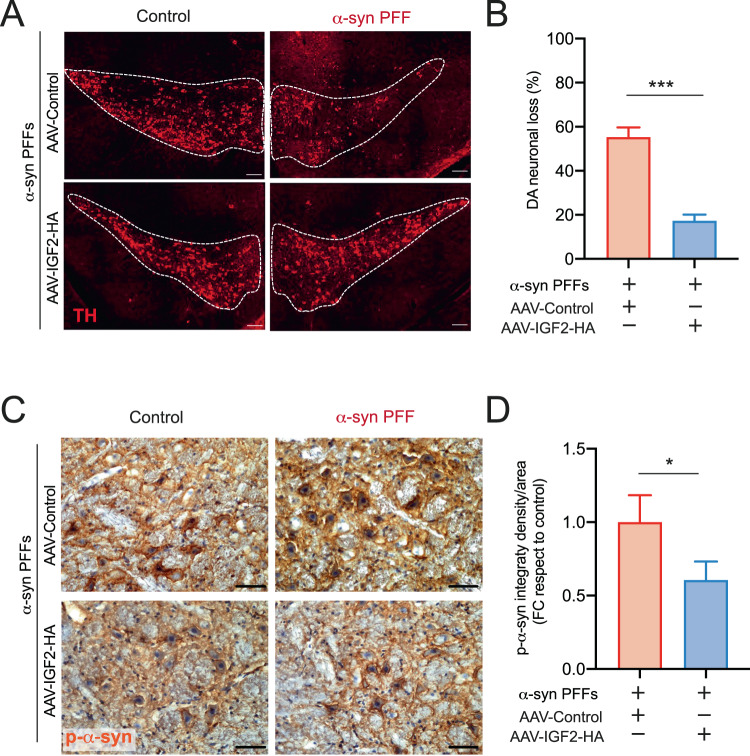
Local delivery of IGF2 into the SNpc prevent dopaminergic neuronal loss and decrease the p-α-syn accumulation in idiopathic PD mice model. **A** At 3 months-old WT animals were injected with α-syn PFF into the right striatum by brain stereotaxis. At 6 weeks after α-syn PFF delivery, the mice were injected with AAV carrying either IGF2 (AAV-IGF2) or empty (AAV-control) into the right SNpc by brain stereotaxis. After 6 weeks, dopaminergic neurons were visualized in midbrain tissue sections by anti-tyrosine hydroxylase (TH) immunostaining (red). Representative images of TH staining in midbrain tissue sections. Scale bar: 50 μm. **B** TH-positive neurons were quantified as the percentage of neurons in the injected side relative to the non-injected (control) in the Substancia Nigra (SN) (left panel). **C** At 3 months-old WT animals were injected with α-syn PFF into the right striatum by brain stereotaxis. At 6 weeks after PFF delivery, the mice were injected with AAV carrying either IGF2 (AAV-IGF2) or empty (AAV-control) into the right SNpc by brain stereotaxis. After 6 weeks, phosphor -α-syn (p-α-syn) accumulation were visualized in midbrain tissue sections by anti-p-α-syn immunostaining (brown). Hematoxylin-Eosin staining (blue/violet) was used to visualize the total cells. Representative images of p-α-syn staining in midbrain tissue sections. Scale bar: 20 μm. **D** p-α-syn positive neurons were quantified using integrated density per area and the percentage of p-α-syn staining on the injected side relative to the non-injected was determine in each condition. FC correspond to fold change. For all experiment mean and SEM were represented for 7 independent experiments. Statistically significant differences were detected by two-tailed unpaired *t* test (***p < 0.001; *p < 0.05).

To determine the possible neuroprotective effects of IGF2 in dopaminergic neurons, we quantified the number of dopaminergic neurons located in the entire SN region through serial sections brain analysis. A global reduction in the rates of dopaminergic neuronal loss was observed by AAV-IGF2 administration into the SNpc region (Fig. 5A, B). We also we evaluated the effect of IGF2 gene therapy in phosphorylated α-syn accumulation into SN region. Remarkably, the expression of IGF2 at the SNpc significantly reduced the content of p-α-syn levels compare to the control mice group (Fig. 5C, D).

**Fig. 6:**
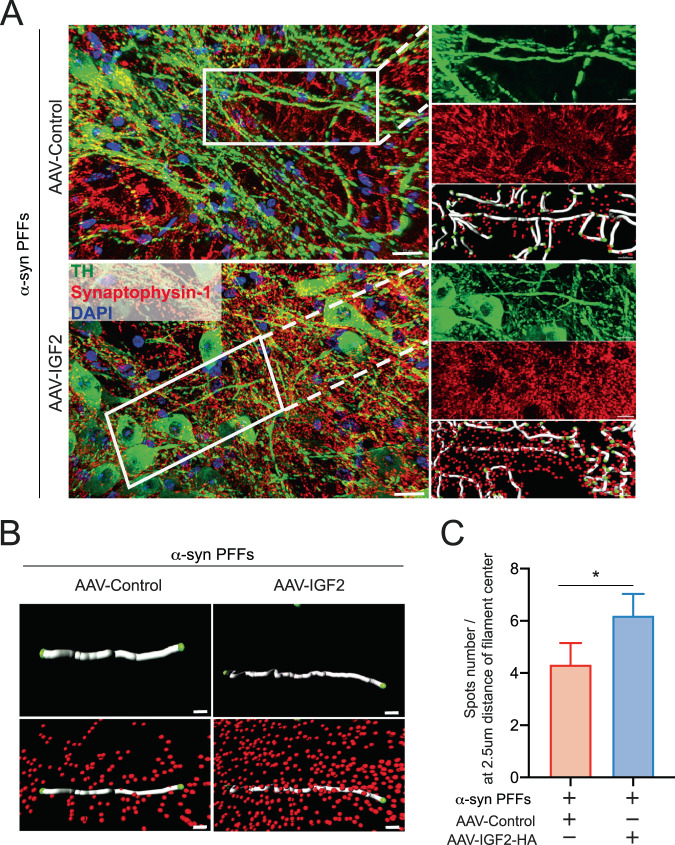
IGF2 treatment rescues dendritic spines loss in PD mice model. **A** Immunofluorescence for dopaminergic neurons were detected using anti-TH antibody, anti-synaptophysin was used as a presynaptic marker. Image represents the selection of analyzed filaments (gray dendrite) and spots (red spots). Scale bar = 10 μm. **B** High magnification to show filaments and spots analyzed. Comparison between AAV-control and AAV-IGF2 conditions. Scale bar = 2 μm. **C** Quantification of number spots present in filaments of 10 μm. Spots present at 2.5 μm from the center of the filament were analyzed. Data represents ~2800 filaments analyzed for AAV-control condition and ~9400 filaments analyzed for AAV-IGF2. Images were analyzed using Imaris software. For all experiment mean and SEM were represented for three independent experiments. Statistically significant differences were detected by two-tailed unpaired *t* test (*p < 0.05).

To explain the possible neuroprotective effect triggers for IGF2 treatment on dopaminergic neuron, we analyzed the number of synaptic spines in SN region. First, we perform immunostaining to label dopaminergic neurons using anti-TH as dopaminergic marker and anti-synaptophysin-1 antibodies as a presynaptic marker (Fig. 6A). Synaptophysin-1 is a calcium-binding and integral membrane glycoprotein present in presynaptic vesicles in neurons, suggesting an involvement in synaptic vesicle endocytosis [55]. We analyzed TH-positive filaments (dopaminergic neurons) and the abundance of synaptophysin-1 positive spots (spines) at 2.5 um of distance from the center of each filament (Fig. 6B). Our results show that IGF2 increases significatively the number of synaptic spines in dopaminergic neurons in comparison with control mice group (Fig. 6C).

Overall, our data suggest a neuroprotective effect of IGF2 treatment in a PD preclinical model based on the intracerebral injection of α-syn PFFs like observed in cellular PD models. The IGF2 neuroprotective effect could be mediated at least by a decrease of α-syn accumulation in dopaminergic neurons and the prevention or delay of synaptic damage triggered by the intracellular load of misfolded α-syn.

## Discussion

Several evidence describes a prion-like behavior of α-syn protein aggregates, resulting in their propagation through the brain [56–58]. The injection of α-syn PFFs into striatum region induces intracellular inclusions in the neuron and spread to others brain regions. This phenomenon is mediate by endocytosis and exocytosis of α-syn PFFs, in a concentration-dependent manner enhancing α-syn PFFs pathology. Additionally, α-syn PFFs incubation in primary neurons led to the activation of both caspase 8, an initiator caspase activated in the extrinsic pathway, and caspase 9, an initiator caspase activated in the intrinsic pathway, suggesting α-syn PFFs triggers apoptotic mechanisms generating dopaminergic neuronal death [57]. Considering this evidence IGF2 treatment reduces the cytotoxicity trigger by α-syn PFF, suggesting a neuroprotective effect possible by reduction of apoptosis activation. This phenomenon could be explained for recently evidence showing the role of endogenous α-syn on the seeding and aggregation, which define the pattern and severity of aggregation and the extent of p-α-syn deposition [59].

In this context, IGF2 deficiency in PD patients could by associate to with a decrease of the control of protein homeostasis by IGF2 signaling and loss of proteostasis could be contributed to increasing the α-syn intracellular load in the Central nervous system [10, 46]. Moreover, our results in cellular PD models indicates that the IGF2 neuroprotective effect is mediate by IGF2 receptor. Cation-independent mannose-6-phosphate/IGF2 receptor is a membrane-bound glycoprotein. The major function of this receptor is trafficking of lysosomal enzymes from the trans-Golgi network to the endosomes and their subsequent transfer to lysosomes for degradation. Also, some proteins may be sorted into vesicles to endocytic recycling compartment from where it could be secreted [60]. This enhance of misfolded protein secretion arise as potential mechanism of IGF2 neuroprotective effect. Additionally, the reduction of the α-syn intracellular levels induced by IGF2 treatments in vivo and in vitro may be explained by an attenuation in the synthesis rate or an increase in degradation mechanisms. IGF2 signaling are involved in activation of degradation pathways such us autophagy by IGF1 receptor activation [61, 62]. However, recent study from our laboratory showed that IGF2 treatment decreases the mHtt aggregates independent of the autophagy or proteasome pathways [10].

On the other hand, IGF2 has been shown to ameliorate synaptic deficits, and cognitive impairments in AD transgenic mice [31, 63, 64]. Since IGF2 can enhance working memory via the IGF2 receptor [18, 65], IGF2 signaling may have a role in regulating both behavior functions and synaptic plasticity and could be also explain the IGF2 neuroprotective effect observed in dopaminergic neuron expose to α-syn PFF in vivo.

The neuronal circuit functionality requires appropriate synaptic connectivity where is essential the morphology, number, and density of the dendritic spines [66, 67]. Also, synaptic degeneration has been involved in many neurological disorders including, Dementia, motor neurons diseases, AD, HD, and PD [68, 69]. For this reason, the prevention of synaptic dysfunction is a key event to maintain the circuit functionality. Here IGF2 treatment showed a positive correlation between motor function recovery, prevention of dopaminergic neuronal loss, decrease of α-syn misfolded levels and synaptic function in dopaminergic neurons in vivo, however, is necessary to demonstrate the possible molecular mechanism underlying of IGF2 signaling.

Presynaptic markers are widely used to evaluate synaptic density, morphology, and functions. One of the most utilized markers for presynaptic proteins is synaptophysin, a major membrane protein found in the membrane of synaptic vesicles representing a ~90–95% localization on synaptic terminals. Its ubiquity at the synapse has led to the use of synaptophysin immunostaining for synaptic quantification [70]. More important the loss of synaptophysin has been describe in neurodegenerative disease such, dementia with Lewy bodies, AD, PD and other [71].

Previous studies shows that α-syn accumulation in PD, MSA and dementia with Lewy Bodies, triggers synaptophysin-1 loss [71]. Additionally, synaptophysin has been describe as presynaptic marker in dopaminergic cell lines such us SHSY5Y cell line [72]. For this reason, results interesting that AAV-IGF2 treatment could prevent the loss of synaptophisin-1 levels associated with the maintenance of synaptic activity in dopaminergic neurons. Moreover, synaptic dysfunction is related to the recruitment of synaptic protein for α-syn aggregates leading to neurotransmitter release impairment and decreases synaptic connections [73, 74]. We could suggest that the protective effect of IGF2 on the loss of dendritic spines could be due to the reduction of the intracellular load of α-syn leads to an increase of neurotransmitter release and a delay in synaptic damage. Importantly, studies provide evidence of the involvement of nuclear factor k-B (NF-kB) signaling in regulation of synaptic plasticity through structural changes, formation and maturation, where IGF2 is directly targeted by NF-kB and identified NF-kB–IGF2–IGF2R signaling axis tightly controlling the number of synaptic connections in hippocampal cultures and remodeling of synaptic connection [75]. Also, IGF2 gene therapy in hippocampus brain region induces synaptic formation and restores spine density and excitatory synaptic transmission in AD mice model [64].

However, it is important to determine the mechanism by which IGF2 would be trigger this neuroprotective effect in dopaminergic neurons. Like our results has been showed the neuroprotective effect of recombinant IGF2 related to oxidative-mitochondrial damage in PD models (MPTP/MPP+) by its interaction with IGF2R. In this pharmacological PD model IGF2 prevented the mitochondrial dysfunction and the activation of nuclear factor (erythroid-derived 2)-like2 (NRF2) [76]. Moreover, recently was described the intranasal recombinant IGF2 administration ameliorated the dopaminergic neuronal loss induced by 6-OHDA models through IGF2R and IGF2R/PI3K/AKT signaling [77].

Overall, the IGF2 treatment prevent dopaminergic neuronal loss by decrease of α-syn aggregates and improve synaptic function and arise as a relevant therapeutic target to prevent or delay the PD progression.

## Materials and methods

### Reagents and DNA plasmids

recombinant IGF2, recombinant mouse and human α-synuclein, and Leu27 peptide were purchased from Sigma. Blocking antibody for IGF2R was purchased from Santa Cruz (sc-25462). Cell media and antibiotics were obtained from Invitrogen (MD, USA). Fetal calf serum was obtained from Hyclone and Sigma. All transfections for plasmids were performed using the Effectene reagent (Qiagen). DNA was purified with Qiagen kits. IGF2 cDNA was obtained from pSPORT6 kindly provided by Dr. Oliver Bracko and subcloned with or without the HA epitope into a pAAV vector. α-synuclein-WT-RFP vectors were provided by Dr. Hiroyoshi Ariga.

### Cell culture and treatment

SN4741 and SHSY5Y cells were obtained from ATCC and maintained in Dulbecco’s modified Eagles medium (DMEM) supplemented with 5% fetal bovine serum. 3 × 10^5^ cells were seeded in 6-well plate and maintained by indicated times in DMEM cell culture media supplemented with 5% bovine fetal serum and non-essential amino acids.

Cortical neurons were obtained from embryonic day 18 mice [78] and infected with AAV particles (1 × 10^9^) at 1 day in vitro. These cells were treated with 1 µg/µl of mouse α-syn PFFs for 72 h.

SN4741 and SHSY5Y cells were treated with 1 µg/ul of recombinant human α-syn PFFs. α-syn PFFs were generated as previously described [57].

### Cell viability analysis

The Sytogreen or propidium iodide (PI) permeability were used to determine cell viability. Sytogreen incorporation into the cell was determine using Fluor Star equipment. PI-positive cell were measurement by microscopy analysis. Cell viability was assessed by the measurement of lactate dehydrogenase (LDH) released from damaged cells in the extracellular media using a Cytotoxicity Detection Kit.

### Western blot and dot blot

Protein aggregation was evaluated by western blot in total cell extracts prepared in 1% Triton X-100 in PBS containing proteases and phosphatases inhibitors (Roche). Protein quantification was performed with the Pierce BCA Protein Assay Kit (Thermo Scientific). For western blot analysis, cells were collected and homogenized in RIPA buffer (20 mM Tris pH 8.0, 150 mM NaCl, 0.1% SDS, 0.5% Triton X-100) containing protease and phosphatase inhibitors (Roche). After sonication, protein concentration was determined in all experiments by micro-BCA assay (Pierce), and 25–100 µg of total protein was loaded onto 8–15% SDS-PAGE minigels (Bio-Rad Laboratories, Hercules, CA) prior transfer onto PVDF membranes. Membranes were blocked using PBS, 0.1% Tween-20 (PBST) containing 5% milk for 60 min at room temperature and then probed overnight with primary antibodies in PBS, 0.02% Tween-20 (PBST) containing 5% skimmed milk. The following primary antibodies and dilutions were used: anti-alpha-Synuclein (BD, Cat. no. 610787), anti-HSP90 1:2000 (Santa Cruz, Cat. no. SC-13119), anti-actin 1:2000 (Santa Cruz, Cat. no. SC-47778) and anti-HA 1:500 (Santa Cruz, Cat. no. SC-805). Bound antibodies were detected with peroxidase-coupled secondary antibodies incubated for 2 h at room temperature and the ECL system.

For dot blot, culture media of each condition were centrifugated for 1000 × g for 3 min and were subjected to vacuum filtration through a 96-well dot blot apparatus (Bio-Rad Laboratories, Hercules, USA) containing a 0.2 μM cellulose acetate membrane (Whatman, GE Healthcare) as described in (Torres et al., 2015). Membranes were then blocked using PBS, 0.1% Tween-20 (PBST) containing 5% milk and incubated with primary antibody at 4 °C overnight. Image quantification was done with the Image Lab software from Bio-Rad.

### Adeno-associated viral vectors

All AAV (serotype 2) vectors were produced by triple transfection of 293 cells using a rep/cap plasmid and pHelper (Stratagene, La Jolla CA, USA), and purified by column affinity chromatography as previously described [8, 79–81]. Also, AAV preparation were produced by Vector Builder Company.

### Animals and surgical procedures

Adult male mice C57BL/6j (3-month-old) were injected with 2 µl of virus AAV-IGF2 or AAV-empty (Control) in the right SNpc, using the following coordinates: AP: −0.29 cm, ML: −0.13 cm, DV: −0.42 cm (according to the atlas of Franklin and Paxinos, Second Edition, 2001), with a 1 µl/min infusion rate. The titer virus used was 1 × 10^8^ viral genomes/μl (VGs) for each of them.

For the generation of PD idiopathic model, recombinant mouse α-syn PFFs were generated as previously described [57]. Briefly, mouse α-syn protein was dissolved in PBS, the pH adjusted to 7.5, subsequently the protein filtered through 100 kDa MW-cut-off filters and incubated with constant agitation (1000 rpm) for 5 days at 37 °C. After incubation the pellet, containing the insoluble fibrils, was separated from the supernatant by ultracentrifugation (100,000 × *g*, 30 min, 4 °C), re-suspended in PBS, and the fibrils fragmented by sonication (5 sec, 20% amplitude, 1× pulse on and 1× pulse off, for four times on ice) to obtain smaller seeds, aliquoted and stored at −80 °C until use. The presence of amyloid-like fibrils was characterized by thioflavin stain. We used 3-month-old male C57BL/6 mice. We injected 5 μg of mouse α-syn PFF in a single point, in the right striatum using the following coordinates: AP: +0.07 cm, ML: −0.17 cm, DV: −0.31 cm (according to the atlas of Franklin and Paxinos Second Edition, 2001).

All experiments were performed in accordance with the guidelines set by the animal care and use committee of the Bioethics at the University Mayor, with approved animal experimentation protocol.

### Motor test

Motor impairment was evaluated by the Beam test which is useful for detecting subtle deficits in motor skills and balance and consists in the record the movement of the mouse in a rod and determining the error of put the paw into the hole or loss of balance [54].

### Tissue preparation and histological analysis

Mice were anesthetized and perfused through the ascending aorta with isotonic saline followed by ice-cold 4% paraformaldehyde in 0.1 M PBS (pH 7.4). Brains were frozen and coronal sections of 25 or 30 μm containing the rostral striatum and midbrain were cut on a cryostat (Leica, Germany). Free-floating midbrain and striatal tissue sections were stained following standard protocols.

For immunohistochemical analysis, sections were incubated overnight at 4 °C in blocking solution with anti-TH (1:2500; Chemicon) or anti-p-a-synuclein (1:500; Bioleagend) antibody and developed with biotinylated secondary anti-rabbit (1:500; Vector Laboratories) and avidin-biotin- peroxidase complex (ABC Elite Kit; Vector Laboratories). For immunofluorescence analysis, sections were incubated overnight at 4 °C in blocking solution with anti-TH (1:1000; Chemicon) and anti-synaptophysin (1:1000; Abcam) antibodies and detected using secondary alexa-488 anti-rabbit and alexa-564 anti-mouse antibodies. Tissue staining were visualized with an inverted microscope Leica DMi8 for scanning complete sections.

The number of TH-positive neurons in the SN region was performed manually by a blind researcher. Results are expressed as the total number of TH-positive neurons per hemisphere. To determine the percentage of TH-positive cell loss in the SNpc of injected mice, the number of dopaminergic cells in the injected and non-injected side was determined by counting in a blinded manner the total number of TH-positive cells in midbrain serial sections containing the entire SNpc (between the AP−0.29 and AP−0.35 cm coordinates)

Results are expressed as the percentage of TH-positive neurons in the injected side compared with the non-injected side. In addition, for striatum denervation quantifications, the images obtained by phase-contrast microscopy from serial sections covering the entire striatum were analyzed using ImageJ software (http://rsb.info.nih.gov/ij/). The total integrated density per hemisphere in the area was quantified. Results are expressed as the percentage of integrated density in the injected side compared with the non-injected side.

### Synaptic spine measurement

Synaptic terminals in SN were labeled by performing immunofluorescence with the use of an anti-synaptophysin antibody as a presynaptic marker. Following acquisition, images were subjected to confocal microscopy. The obtained images were then processed using the Huygens Professional software (Scientific Volume Imaging, The Netherlands, version [latest version]), applying the Classic Maximum Likelihood Estimation algorithm for image deconvolution. The deconvolved images were exported in the Huygens H4 format for subsequent analysis.

In the next step, we utilized Imaris software (Bitplane, Switzerland, version [latest version]) for neuron and synapse identification. The Filament Tracer tool was employed to recognize and measure neurite lengths, with the parameter of diameter being meticulously adjusted to match our samples. The Spot Detector tool was subsequently used for synaptic termini identification, with an estimated diameter for spots detection set at 0.9 μm.

The spots were selected based on their distance from the filament, employing a custom-made function within the Imaris software. Only the spots located within a maximum distance of 2.5 μm from the filament’s center were deemed positive and included in further analysis. This procedure was conducted in three-dimensional space to better represent the complex architecture of the dendritic trees.

In the final stage, data were normalized by the total length of dendrites, and are presented as the number of spines per 10 microns of dendrite. We processed ~2800 filaments for the control condition (AAV-GFP) and ~9400 filaments for the treated condition (AAV-IGF2).

### Statistical analysis

Results were statistically compared using the student’s *t* test was performed for unpaired or paired groups. Where pertinent two-way ANOVA for unpaired groups followed by multiple comparison post tests (Bonferroni Test). A *p* value of <0.05 was considered significant (*p < 0.05, **p < 0.01, ***p < 0.001)

### Supplementary information

Original data files

## Data Availability

The materials described in the manuscript, including all relevant raw data, will be freely available to any researcher wishing to use them for non-commercial purposes, without breaching participant confidentiality.
